# Supplementary material for: The Role of Fibroblast Activation Protein Inhibitor Positron Emission Tomography in Inflammatory and Infectious Diseases: An Updated Systematic Review
Source: Pharmaceuticals (Basel). 2024 May 31;17(6):716. doi: 10.3390/ph17060716 (PMC11206476; doi:10.3390/ph17060716)
Supplement: Supplementary file 1 [file pharmaceuticals-17-00716-s001.zip › pharmaceuticals-3037689-supplementary/Supplemental Table S2.pdf]

Table S2. list of excluded studies with reasons.

Excluded Studies	Reason for exclusion
Wang Y, Liu Y, Geng H, Zhang W. Advancements in theranostic applications: exploring the role of fibroblast activation protein inhibition tracers in enhancing thyroid health assessment. EJNMMI Res. 2023 Dec 22;13(1):109. doi:10.1186/s13550-023-01060-8. PMID: 38129604; PMCID: PMC10739649.	Review in the field of interest
Zhang Z, Hou W, Pan G, Zuo C, Cheng C. Elevated 68 Ga-FAPI-04 Activity Due to Staphylococcus aureus Intracranial Infection. Clin Nucl Med. 2024 Jan 1;49(1):e6-e7. doi: 10.1097/RLU.0000000000004958. Epub 2023 Nov 16. PMID:37976533.	Case report in the field of interest
Chen X, Liu M, Yang L, Shu Q, Cai L. 68 Ga-FAPI and 18 F-FDG Uptake in a Patient With Esophageal Candida Infection. Clin Nucl Med. 2024 Jan 1;49(1):83-85. doi: 10.1097/RLU.0000000000004946. Epub 2023 Nov 16. PMID:37976435.	Case report in the field of interest
Liu Z, Zhou H, Li P, Wang Z, Tu T, Ezzi SHA, Kota VG, Hasan Abdulla MHA, Alhaskawi A, Dong Y, Huang Y, Dong M, Su X, Lu H. Fibroblast Activation Protein-Targeted PET/CT With Al¹⁸F-NODA-FAPI-04 for In Vivo Imaging of Tendon Healing in Rat Achilles Tendon Injury Models. Am J Sports Med. 2023 Dec;51(14):3790-3801. doi: 10.1177/03635465231208843. Epub 2023 Nov 17. PMID:37975494.	Original study not in the field of interest
Xie Y, Tang W, Ma J, Wang Y, Chen Y. Elevated 68 Ga-FAPI Activity in Klebsiella pneumoniae Invasion Syndrome. Clin Nucl Med. 2024 Jan 1;49(1):89-90. doi: 10.1097/RLU.0000000000004925. Epub 2023 Nov 7. PMID: 37937955.	Case report in the field of interest
Li T, Liu Z, Zhang Z, Zhang J, Zhao X. Multiple Intrahepatic Inflammatory Myofibroblastic Tumor on 68 Ga-FAPI and 18 F-FDG PET/CT. Clin Nucl Med. 2023 Dec 1;48(12):e614-e616. doi: 10.1097/RLU.0000000000004924. Epub 2023 Oct 23. PMID:37883213.	Case report in the field of interest
Lavis P, Pingitore J, Doumont G, Garabet A, Van Simaeys G, Lacroix S, Passon N, Van Heymbeek C, De Maeseneire C, Allard J, Collin A, Huaux F, Decaestecker C, Salmon I, Goldman S, Cardozo AK, Bondue B. Usefulness of FAPα assessment in bronchoalveolar lavage as a marker of fibrogenesis: results of a preclinical study and first report in patients with idiopathic pulmonary fibrosis. Respir Res. 2023 Oct 25;24(1):254. doi: 10.1186/s12931-023-02556-6. PMID: 37880678;	Preclinical study in the field of interest

PMCID: PMC10601150.	
Hoppner J, van Genabith L, Hielscher T, Heger U, Sperling L, Colbatzky T, Gutjahr E, Lang M, Pausch T, Spektor AM, Glatting FM, Liermann J, Hackert T, Kratochwil C, Giesel FL, Haberkorn U, Röhrich M. Comparison of early and late ⁶⁸Ga-FAPI-46-PET in 33 patients with possible recurrence of pancreatic ductal adenocarcinomas. Sci Rep. 2023 Oct 19;13(1):17848. doi:10.1038/s41598-023-43049-2. PMID: 37857656; PMCID: PMC10587145.	Original study not in the field of interest
Cai J, Xu W, Meng T, Pang Y, Chen H. Acute Appendicitis Complicated by Septic Thrombophlebitis of the Portal Vein Shown by 18 F-FDG and 68 Ga-FAPI-46 PET/CT. Clin Nucl Med. 2023 Nov 1;48(11):997-999. doi: 10.1097/RLU.0000000000004830. Epub 2023 Sep 18. PMID: 37796180.	Case report in the field of interest
Roshanravan V, Sadeghi R, Zakavi SR, Norouzbeigi N, Aghaee A. Unilateral Chronic Parotitis With 68 Ga-DOTA-FAPI-04 PET/CT and 18 F-FDG PET/CT Imaging in a Case of a Patient With Papillary Thyroid Carcinoma. Clin Nucl Med. 2023 Dec 1;48(12):e577-e579. doi: 10.1097/RLU.0000000000004884. Epub 2023 Sep 23. PMID: 37756475.	Case report in the field of interest
Hirata K, Kamagata K, Ueda D, Yanagawa M, Kawamura M, Nakaura T, Ito R, Tatsugami F, Matsui Y, Yamada A, Fushimi Y, Nozaki T, Fujita S, Fujioka T, Tsuboyama T, Fujima N, Naganawa S. From FDG and beyond: the evolving potential of nuclear medicine. Ann Nucl Med. 2023 Nov;37(11):583-595. doi: 10.1007/s12149-023-01865-6. Epub 2023 Sep 25. PMID: 37749301.	Review in the field of interest
Demmert TT, Pomykala KL, Lanzafame H, Pabst KM, Lueckerath K, Siveke J, Umutlu L, Hautzel H, Hamacher R, Herrmann K, Fendler WP. Oncologic Staging with ⁶⁸Ga-FAPI PET/CT Demonstrates a Lower Rate of Nonspecific Lymph Node Findings Than ¹⁸F-FDG PET/CT. J Nucl Med. 2023 Dec 1;64(12):1906-1909. doi: 10.2967/jnumed.123.265751. PMID: 37734836.	Original study not in the field of interest
Lyu Z, Han W, Zhang Q, Zhao H, Liu S, Wang Y, He J, Zhao C, Tian L, Fu P. Clinical application of Al¹⁸F-NOTA-FAPI PET/CT in diagnosis and TNM staging of pancreatic adenocarcinoma, compared to ¹⁸F-FDG. Cancer Imaging. 2023 Sep 12;23(1):86. doi: 10.1186/s40644-023-00596-1. PMID: 37700343; PMCID: PMC10496317	Original study not in the field of interest
Shangguan C, Yang C, Shi Z, Miao Y, Hai W, Shen Y,	Original study not in the field of interest

Qu Q, Li B, Mi J. ⁶⁸Ga-FAPI-04 Positron Emission Tomography Distinguishes Malignancy From ¹⁸F-FDG-Avid Colorectal Lesions. Int J Radiat Oncol Biol Phys. 2024 Jan 1;118(1):285-294. doi: 10.1016/j.ijrobp.2023.08.019. Epub 2023 Aug 25. PMID: 37634891	
Li X, Ma W, Wang M, Quan Z, Zhang M, Ye J, Li G, Zhou X, Ma T, Wang J, Yang W, Nie Y, Wang J, Kang F. ⁶⁸Ga-FAPI-04 PET for Surveillance of Anastomotic Recurrence in Postoperative Patients with Gastrointestinal Cancer: a Comparative Study with ¹⁸F-FDG PET. Mol Imaging Biol. 2023 Oct;25(5):857-866. doi: 10.1007/s11307-023-01835-4. Epub 2023 Jul 5. PMID: 37407745.	Original study not in the field of interest
Wang AB, Feng LJ, Ni PP, He LM, Li HM, Zhang W. [Causes of False-Positive Results in ⁶⁸Ga-Labeled Fibroblast Activation Protein Inhibitor PET/CT Imaging]. Zhongguo Yi Xue Ke Xue Yuan Xue Bao. 2023 Jun;45(3):440-444. Chinese. doi: 10.3881/j.issn.1000-503X.15221. PMID: 37407532.	Original study in the field of interest not in English
Rasinski P, Af Burén S, Holstensson M, Nilsson T, Loizou L, Tran TA, Sparrelid E, Löhr JM, Axelsson R. Tumor Characterization by [⁶⁸Ga]FAPI-46 PET/CT Can Improve Treatment Selection for Pancreatic Cancer Patients: An Interim Analysis of a Prospective Clinical Trial. J Nucl Med. 2023 Aug;64(8):1232-1237. doi: 10.2967/jnumed.123.265481. Epub 2023 Jun 22. PMID: 37348917.	Original study not in the field of interest
Tatar G, Beyhan E, Erol Fenercioğlu Ö, Sevindir İ, Ergül N, Çermik TF. ⁶⁸Ga-FAPI-04 PET/CT Findings in Patients with Liver Cirrhosis. Mol Imaging Radionucl Ther. 2023 Jun 20;32(2):146-149. doi: 10.4274/mirt.galenos.2022.80774. PMID: 37337827; PMCID: PMC10284187	Case series in the field of interest
Ou L, Zhou Y, Sun R, Zhang C, Chen X. Increased 68 Ga-FAPI Activity in Hepatic Inflammatory Myofibroblastoma. Clin Nucl Med. 2023 Jun 1;48(6):522-524. doi: 10.1097/RLU.0000000000004662. Epub 2023 Apr 24. PMID: 37083628.	Case report in the field of interest
Sun G, Zou R, Yao L, Zuo C. 68Ga-FAPI-04 PET/MR Versus 18F-FDG PET/CT in the Detection of Ovarian Cancer. Clin Nucl Med. 2023 Jun 1;48(6):525-527. doi: 10.1097/RLU.0000000000004656. PMID: 37133512.	Case report in the field of interest
Zhang Q, Lin X, Wang W, Zhang X, Lü M, Shao Z, Shi D, Zhang R, Shi H, Zhang Y, Pan J, Song G, Cheng K,	Preclinical study in the field of interest

Ge L, Wang L, Han J. Evaluation of ¹⁸F-FAPI-04 Imaging in Assessing the Therapeutic Response of Rheumatoid Arthritis. Mol Imaging Biol. 2023 Aug;25(4):630-637. doi:10.1007/s11307-023-01817-6. Epub 2023 Apr 5. PMID: 37020126.	
Sviridenko A, di Santo G, Virgolini I. Imaging Fibrosis. PET Clin. 2023 Jul;18(3):381-388. doi: 10.1016/j.cpet.2023.02.004. Epub 2023 Mar 27. PMID:36990946.	Review in the field of interest
Liu W, Gong W, Yang X, Xu T, Chen Y. Increased FAPI Activity in Pulmonary Tuberculosis. Clin Nucl Med. 2023 Feb 1;48(2):188-189. doi: 10.1097/RLU.0000000000004498. Epub 2022 Dec 7. PMID: 36607369	Case report in the field of interest
Liu Y, Guo C, Chen L, Huang Z. 68Ga-FAPI PET/CT in a Patient With Statin-Induced Rhabdomyolysis. Clin Nucl Med. 2023 Jan 1;48(1):77-78. doi: 10.1097/RLU.0000000000004418. Epub 2022 Sep 28. PMID: 36469065	Case report in the field of interest
Mu X, Zhu Z, Fu W. Increased 18F-FAPI PET/CT Uptake in a Case of Brucellosis of Lumbar Vertebral Body. Clin Nucl Med. 2023 Jan 1;48(1):75-76. doi: 10.1097/RLU.0000000000004467. Epub 2022 Oct 26. PMID: 36469064	Case report in the field of interest
Cheung SK, Chen S, Wong YH, Wu KK, Ho CL. Diagnosis of Seronegative Rheumatoid Arthritis by 68 Ga-FAPI PET/CT. Nucl Med Mol Imaging. 2023 Feb;57(1):44-45. doi: 10.1007/s13139-022-00779-x. Epub 2022 Oct 20. PMID: 36643942; PMCID: PMC9832189	Case report in the field of interest
Gong W, Qiu S, Zheng S, Liu W, Zhang C. 68Ga-FAPI PET/CT Imaging of Intracranial Syphilitic Gumma: Comparison to 18F-FDG PET/CT. Clin Nucl Med. 2023 Mar 1;48(3):273-275. doi: 10.1097/RLU.0000000000004479. Epub 2022 Dec 24. PMID: 36723888	Case report in the field of interest
Meetschen M, Sandach P, Darwiche K, Theegarten D, Moter A, Schaarschmidt BM, Herrmann K, Fendler WP, Hautzel H, Opitz M. Rabbit fever: granulomatous inflammation by Francisella tularensis mimics lung cancer in dual tracer ¹⁸FDG and ⁶⁸Ga-FAPI PET/CT. Eur J Nucl Med Mol Imaging. 2023 Jul;50(8):2567-2569. doi: 10.1007/s00259-023-06175-7. Epub 2023 Mar 13. PMID: 36907901; PMCID: PMC10008710.	Case report in the field of interest

Treglia G, Albano D. FAPI PET/CT in infectious, inflammatory, and rheumatological diseases: "watch it like a hawk" or "one swallow does not make a summer"? Eur J Nucl Med Mol Imaging. 2023 Jun;50(7):1848-1850. doi:10.1007/s00259-023-06179-3. PMID: 36872391; PMCID: PMC9986035.	Editorial in the field of interest
Bentestuen M, Al-Obaydi N, Zacho HD. FAPI-avid nonmalignant PET/CT findings: An expedited systematic review. Semin Nucl Med. 2023 Sep;53(5):694-705. Doi: 10.1053/j.semnuclmed.2023.02.001. Epub 2023 Feb 20. PMID: 36813670.	Review not in the field of interest
Sellmann T, Staak F, Maurer C, Rott G, Witzke O, Fendler W, Schildhaus HU, Podleska LE, Herrmann K, Rischpler C. Incidental Detection of a Tenosynovial Giant Cell Tumor of the Thigh on [⁶⁸Ga]Ga-FAPI PET/CT: Presentation of an Unusual Case. J Nucl Med. 2023 Jul;64(7):1162-1163. Doi: 10.2967/jnumed.118.207761. Epub 2023 Jan 26. PMID: 36702554.	Case report not in the field of interest
Jagtap RR, Savale SV, Khajindar GS, Solav SV. Localization of Pyelonephritis by ⁶⁸Ga-FAPI PET CT. Asia Ocean J Nucl Med Biol. 2023;11(1):85-88. doi: 10.22038/AOJNMB.2022.64168.1450. PMID: 36619193; PMCID: PMC9803616.	Case report in the field of interest
Qi N, Wang H, Wang H, Ren S, You Z, Chen X, Guan Y, Xie F, Hua F, Zhao J. Non-tumoral uptake of ⁶⁸Ga-FAPI-04 PET: A retrospective study. Front Oncol. 2022 Dec 1;12:989595. doi: 10.3389/fonc.2022.989595. PMID: 36531015; PMCID: PMC9751966.	Original article not in the field of interest
Wang Y, Wang R, Geng L, Li Q, Qi E, Shi Y, Wang Y, Zheng Q, Zhang G, Chen J, Tian J. Different uptake patterns of ⁶⁸Ga-FAPI in aseptic loosening and periprosthetic joint infection of hip arthroplasty: A case series and literature review. Front Med (Lausanne). 2022 Nov 24;9:1014463. doi: 10.3389/fmed.2022.1014463. PMID: 36507508; PMCID: PMC9729767.	Case series in the field of interest
Kirienko M, Erba PA, Chiti A, Sollini M. Hybrid PET/MRI in Infection and Inflammation: An Update About the Latest Available Literature Evidence. Semin Nucl Med. 2023 Jan;53(1):107-124. doi: 10.1053/j.semnuclmed.2022.10.005. Epub 2022 Nov 8. PMID: 36369091.	Review not in the field of interest
Glatting FM, Hoppner J, Kauczor HU, Huber PE, Kratochwil C, Giesel FL, Haberkorn U, Röhrich M. Subclass Analysis of Malignant, Inflammatory and	Original article not in the field of interest

Degenerative Pathologies Based on Multiple Timepoint FAPI-PET Acquisitions Using FAPI-02, FAPI-46 and FAPI-74. Cancers (Basel). 2022 Oct 28;14(21):5301. doi:10.3390/cancers14215301. PMID: 36358720; PMCID: PMC9656977.	
Zhang J, He Q, Jiang S, Li M, Xue H, Zhang D, Li S, Peng H, Liang J, Liu Z, Rao S, Wang J, Zhang R, Zhang L. [¹⁸F]FAPI PET/CT in the evaluation of focal liver lesions with [¹⁸F]FDG non-avidity. Eur J Nucl Med Mol Imaging. 2023 Feb;50(3):937-950. doi: 10.1007/s00259-022-06022-1. Epub 2022 Nov 8. PMID: 36346437.	Original article not in the field of interest
Xu T, Zhang Y, Wu R, Ding H, Chen Y. 68Ga-DOTA-FAPI-04 PET/CT Imaging in a Case of Radioactive Iodine-Induced Chronic Parotitis. Clin Nucl Med. 2022 Dec 1;47(12):1082-1083. doi: 10.1097/RLU.0000000000004416. Epub 2022 Sep 28. PMID:36342796.	Case report in the field of interest
Li X, Zhao Q, Luo Q, Chen L, Huang Z. The Manifestation of a Patient With Myelofibrosis in 68Ga-DOTA-FAPI-04 PET/CT Mimicking "Super Bone Imaging". Clin Nucl Med. 2022 Dec 1;47(12):1056-1058. doi: 10.1097/RLU.0000000000004427. Epub 2022 Sep 30. PMID: 36342793.	Case report not in the field of interest
Li H, Kong Z, Xiang Y, Zheng R, Liu S. The role of PET/CT in radiotherapy for nasopharyngeal carcinoma. Front Oncol. 2022 Oct 21;12:1017758. doi: 10.3389/fonc.2022.1017758. PMID: 36338692; PMCID: PMC9634754.	Case report not in the field of interest
Vorster M. Gallium-68 Labelled Radiopharmaceuticals for Imaging Inflammatory Disorders. Semin Nucl Med. 2023 Mar;53(2):199-212. Doi: 10.1053/j.semnuclmed.2022.08.005. Epub 2022 Oct 19. PMID: 36270829.	Review in the field of interest
Schmidkonz C, Kuwert T, Atzinger A, Cordes M, Schett G, Ramming A, Götz T. Fibroblast Activation Protein Inhibitor Imaging in Nonmalignant Diseases: A New Perspective for Molecular Imaging. J Nucl Med. 2022 Dec;63(12):1786-1792. doi: 10.2967/jnumed.122.264205. Epub 2022 Sep 15. PMID: 36109182.	Review in the field of interest
Dendl K, Koerber SA, Tamburini K, Mori Y, Cardinale J, Haberkorn U, Giesel FL. Advancement and Future Perspective of FAPI PET/CT In Gynecological Malignancies. Semin Nucl Med. 2022 Sep;52(5):628-634. doi:10.1053/j.semnuclmed.2022.04.002. Epub 2022 Jul 13. PMID: 35842334.	Review not in the field of interest
Hotta M, Rieger AC, Jafarvand MG, Menon N, Farolfi A, Benz MR, Calais J. Non-oncologic incidental uptake	Review in the field of interest

on FAPI PET/CT imaging. Br J Radiol. 2023 Feb;96(1142):20220463. doi: 10.1259/bjr.20220463. Epub 2022 Jul 13. PMID: 35776566; PMCID: PMC9975522.	
Wang Y, Li Y, Han L, Wang J, Zhang C, Qi E, Zhang D, Zhang X, Huan Y, Tian J. ¹⁸F-FDG and ⁶⁸ Ga-FAPI PET/CT for the evaluation of periprosthetic joint infection and aseptic loosening in rabbit models. BMC Musculoskelet Disord. 2022 Jun 20;23(1):592. doi: 10.1186/s12891-022-05537-w. PMID: 35725436; PMCID: PMC9208226.	Preclinical study in the field of interest
Glatting FM, Hoppner J, Liew DP, van Genabith A, Spektor AM, Steinbach L, Hubert A, Kratochwil C, Giesel FL, Dendl K, Rathke H, Kauczor HU, Huber PE, Haberkorn U, Röhrich M. Repetitive Early ⁶⁸Ga-FAPI PET Acquisition Comparing ⁶⁸Ga-FAPI-02, ⁶⁸Ga-FAPI-46, and ⁶⁸Ga-FAPI-74: Methodologic and Diagnostic Implications for Malignant, Inflammatory/Reactive, and Degenerative Lesions. J Nucl Med. 2022 Dec;63(12):1844-1851. doi: 10.2967/jnumed.122.264069. Epub 2022 May 26. PMID: 35618480; PMCID: PMC9730916.	Original article not in the field of interest
Lan L, Zhang S, Xu T, Liu H, Wang W, Feng Y, Wang L, Chen Y, Qiu L. Prospective Comparison of ⁶⁸Ga-FAPI versus ¹⁸F-FDG PET/CT for Tumor Staging in Biliary Tract Cancers. Radiology. 2022 Sep;304(3):648-657. doi: 10.1148/radiol.213118. Epub 2022 May 17. PMID: 35579524.	Original article not in the field of interest
Kuwert T, Schmidkonz C, Prante O, Schett G, Ramming A. FAPI PET Opens a New Window to Understanding Immune-Mediated Inflammatory Diseases. J Nucl Med. 2022 Aug;63(8):1136-1137. doi: 10.2967/jnumed.122.263922. Epub 2022 Apr 7. PMID: 35393350.	Editorial in the field of interest
Af Burén S, Tran TA, Klevebro F, Holstensson M, Axelsson R. A 68Ga-FAPI-46 PET/CT Imaging Pitfall in Assessing Residual Gastric Cancer Early After Chemotherapy. Clin Nucl Med. 2022 Jul 1;47(7):644-645. doi:10.1097/RLU.0000000000004143. Epub 2022 Mar 31. PMID: 35353747.	Case report not in the field of interest
Zhang Z, Jia G, Pan G, Cao K, Yang Q, Meng H, Yang J, Zhang L, Wang T, Cheng C, Zuo C. Comparison of the diagnostic efficacy of ⁶⁸ Ga-FAPI-04 PET/MR and ¹⁸F-FDG PET/CT in patients with pancreatic cancer. Eur J Nucl Med Mol Imaging. 2022 Jul;49(8):2877-2888. doi: 10.1007/s00259-022-05729-5. Epub 2022 Mar 4. PMID: 35243518.	Original article not in the field of interest

Li M, Younis MH, Zhang Y, Cai W, Lan X. Clinical summary of fibroblast activation protein inhibitor-based radiopharmaceuticals: cancer and beyond. Eur J Nucl Med Mol Imaging. 2022 Jul;49(8):2844-2868. doi:10.1007/s00259-022-05706-y. Epub 2022 Jan 31. Erratum in: Eur J Nucl Med Mol Imaging. 2022 May 6;: PMID: 35098327; PMCID: PMC9232873.	Review not in the field of interest
Wang Y, Liu H, Yao S, Guan Z, Li Q, Qi E, Li X, Zhang J, Tian J. Using 18F-flurodeoxyglucose and 68Ga-fibroblast activation protein inhibitor PET/CT to evaluate a new periprosthetic joint infection model of rabbit due to Staphylococcus aureus. Nucl Med Commun. 2022 Feb 1;43(2):232-241. doi: 10.1097/MNM.0000000000001495. PMID: 35022379.	Preclinical study in the field of interest
Gong W, Yang X, Mou C, Liu H, Zhang C. Bone Tuberculous Granulomatous Inflammation Mimicking Malignancy on 68Ga-FAPI PET/CT. Clin Nucl Med. 2022 Apr 1;47(4):348-349. doi: 10.1097/RLU.0000000000003990. PMID: 35020664.	Case report in the field of interest
Wang Y, Yang X, Tian M, Lv H, Liu H. Orbital Granulomatous Inflammation Mimicking Malignancy on 68Ga-FAPI PET/CT. Clin Nucl Med. 2022 Apr 1;47(4):380-381. doi: 10.1097/RLU.0000000000003982. PMID: 35020645.	Case report in the field of interest
Kuyumcu S, Sanli Y, Subramaniam RM. Fibroblast-Activated Protein Inhibitor PET/CT: Cancer Diagnosis and Management. Front Oncol. 2021 Nov 11;11:758958. doi: 10.3389/fonc.2021.758958. PMID: 34858834; PMCID: PMC8632139.	Review not in the field of interest
Dendl K, Koerber SA, Kratochwil C, Cardinale J, Finck R, Dabir M, Novruzov E, Watabe T, Kramer V, Choyke PL, Haberkorn U, Giesel FL. FAP and FAPI-PET/CT in Malignant and Non-Malignant Diseases: A Perfect Symbiosis? Cancers (Basel). 2021 Sep 30;13(19):4946. doi: 10.3390/cancers13194946. PMID: 34638433; PMCID:PMC8508433.	Review not in the field of interest
Lan L, Liu H, Wang Y, Deng J, Peng D, Feng Y, Wang L, Chen Y, Qiu L. The potential utility of [⁶⁸Ga]Ga-DOTA-FAPI-04 as a novel broad-spectrum oncological and non-oncological imaging agent-comparison with [¹⁸F]FDG. Eur J Nucl Med Mol Imaging. 2022 Feb;49(3):963-979. doi:10.1007/s00259-021-05522-w. Epub 2021 Aug 19. PMID: 34410435.	Original article not in the field of interest
Zhao L, Pang Y, Sun L, Lin Q, Chen H. Increased 68Ga-FAPI Uptake in the Pulmonary Cryptococcus and the Postradiotherapy Inflammation. Clin Nucl Med. 2022 Mar 1;47(3):243-245. doi:	Case report in the field of interest

10.1097/RLU.0000000000003873. PMID: 34406184.	
Wu J, Qiu L, Wang Y, Zhang C. Dermatomyositis on 68Ga-FAPI PET/CT in a Patient With Nasopharyngeal Carcinoma. Clin Nucl Med. 2022 Feb 1;47(2):149-150. doi: 10.1097/RLU.0000000000003809. PMID: 34284484.	Case report not in the field of interest
Zhang Z, Jiang H, Zhang L, Cheng C, Zuo C. 18 F-FDG and 68 Ga-FAPI-04 PET/CT Imaging of a Case With Immunoglobulin G4-Related Disease Presenting as Hepatic Masses. Clin Nucl Med. 2022 Dec 1;47(12):1092-1094. doi: 10.1097/RLU.0000000000004377. Epub 2022 Sep 9. PMID: 36083160	Case report in the field of interest
Zhang Z, Liu Y, Zhang L, Cheng C, Zuo C. Renal Pelvis Immunoglobulin G4-Related Disease Mimicking Malignant Tumor: A Case of 18 F-FDG and 68 Ga-FAPI PET/CT Imaging. Clin Nucl Med. 2022 Sep 1;47(9):815-816. doi: 10.1097/RLU.0000000000004292. Epub 2022 May 27. PMID: 35619197	Case report in the field of interest
Zheng J, Lin K, Zheng S, Yao S, Miao W. 68Ga-FAPI and 18F-PET/CT Images in Intestinal Tuberculosis. Clin Nucl Med. 2022 Mar 1;47(3):239-240. doi: 10.1097/RLU.0000000000003917. PMID: 34619704	Case report in the field of interest
Xu T, Huang Y, Zhao Y, Wang P, Chen Y. 68Ga-DOTA-FAPI-04 PET/CT Imaging in a Case of SAPHO Syndrome. Clin Nucl Med. 2022 Mar 1;47(3):246-248. doi: 10.1097/RLU.0000000000003901. PMID: 34560692	Case report in the field of interest
Yang X, You Z, Gong W, Chen Y, Liu H. Increased 68Ga-FAPI Uptake in Facet Joint Osteoarthritis in a Patient With Esophageal Cancer. Clin Nucl Med. 2022 Apr 1;47(4):342-343. doi: 10.1097/RLU.0000000000003957. PMID: 35020666	Case report in the field of interest
Yang X, You Z, Mou C, Hu Z, Liu H. Esophagitis Mimicking Esophageal Cancer on 68Ga-FAPI PET/CT. Clin Nucl Med. 2022 Mar 1;47(3):279-280. doi: 10.1097/RLU.0000000000003907. PMID: 34653055	Case report in the field of interest
Yang X, Huang Y, Mou C, Liu H, Chen Y. Chronic Colitis Mimicking Malignancy on 68Ga-FAPI PET/CT. Clin Nucl Med. 2022 Feb 1;47(2):159-160. doi: 10.1097/RLU.0000000000003793. PMID: 34238800	Case report in the field of interest
Tang W, Wu J, Yang S, Wang Q, Chen Y. Organizing Pneumonia With Intense 68Ga-FAPI Uptake Mimicking Lung Cancer on 68Ga-FAPI PET/CT. Clin	Case report in the field of interest

Nucl Med. 2022 Mar 1;47(3):223-225. doi: 10.1097/RLU.0000000000003855. PMID: 34392279	
Telo S, Farolfi A, Castellucci P, Antonacci F, Solli P, Mosconi C, Fanti S, Agosti R, Morigi JJ, Nanni C. A case of [68Ga]Ga-FAPI-46-avid and [18F]F-FDG-negative COVID-19 pneumonia sequelae. Eur J Nucl Med Mol Imaging. 2022 Jun;49(7):2452-2453. doi: 10.1007/s00259-022-05720-0. Epub 2022 Feb 18. PMID: 35179626; PMCID: PMC8854481	Case report in the field of interest
Gong W, Fu M, Zhang Y, Yang X, Zhang C. Progressive Multifocal Leukoencephalopathy Mimicking Malignancy on 68Ga-FAPI PET/CT: Potential Advantages of FAPI. Clin Nucl Med. 2022 May 1;47(5):430-432. doi: 10.1097/RLU.0000000000003976. PMID: 35020663	Case report in the field of interest
Dong A, Zhang Z, Zhang L, Cheng C, Zuo C. 68 Ga-FAPI-04 Versus 18 F-FDG PET/CT in a Case of Peutz-Jeghers Syndrome. Clin Nucl Med. 2022 Aug 1;47(8):725-727. doi: 10.1097/RLU.0000000000004113. Epub 2022 Feb 28. PMID: 35234198	Case report in the field of interest
Deng Y, Wu J, Xu C, Zhang W. Primary Solitary Tuberculosis in the Hepatic Round Ligament Detected by 68Ga-FAPI PET/CT. Clin Nucl Med. 2022 May 1;47(5):e414-e416. doi: 10.1097/RLU.0000000000004122. PMID: 35293356	Case report in the field of interest
Alçın G, Tatar G, Şahin R, Baloğlu MC, Çermik TF. Peritoneal Tuberculosis Mimicking Peritoneal Carcinomatosis on 68 Ga-FAPI-04 and 18 F-FDG PET/CT. Clin Nucl Med. 2022 Aug 1;47(8):e557-e558. doi: 10.1097/RLU.0000000000004174. Epub 2022 Apr 5. PMID: 35384886.	Case report in the field of interest
Chen Z, Xue Q, Yang Y, Shun H, Miao W. 68Ga-FAPI and 18F-FDG PET/CT Images of a Patient With Rosai-Dorfman Disease With Liver Involvement. Clin Nucl Med. 2022 Dec 1;47(12):1079-1081. doi: 10.1097/RLU.0000000000004367. Epub 2022 Aug 12. PMID: 36342795	Case report in the field of interest
Erol Fenercioğlu Ö, Beyhan E, Ergül N, Arslan E, Çermik TF. 18F-FDG PET/CT and 68Ga-FAPI-4 PET/CT Findings of Bilateral Knee Osteoarthritis in a Patient With Uveal Malignant Melanoma. Clin Nucl Med. 2022 Feb 1;47(2):e144-e146. doi: 10.1097/RLU.0000000000003854. PMID: 34319962	Case report in the field of interest

Fu L, Zhou W. Active uptake of [18F]F-FAPI-42 in eosinophilic gastrointestinal disorder. Eur J Nucl Med Mol Imaging. 2022 Dec 1. doi: 10.1007/s00259-022-06055-6. Epub ahead of print. PMID: 36450937	Case report in the field of interest
Guo W, Chen H. 68Ga FAPI PET/MRI in Cardiac Amyloidosis. Radiology. 2022 Apr;303(1):51. doi: 10.1148/radiol.211951. Epub 2021 Dec 21. PMID: 34931860.	Case report in the field of interest
Ni M, Xie Q, Zhu X, Lv W. Relapsing polychondritis revealed by 18F-FDG and Al 18F-NOTA-FAPI-04 PET/CT. Hell J Nucl Med. 2022 Jan-Apr;25(1):108-110. doi: 10.1967/s002449912441. Epub 2022 Apr 8. PMID: 35388809.	Case report in the field of interest
Sun R, Huang Z, Wei J, Zeng C, Chen X. 68Ga-FAPI and 18F-FDG PET/CT Findings in a Patient With Pancreatic Tuberculosis Mimicking Malignant Tumor. Clin Nucl Med. 2022 Jul 1;47(7):653-654. doi: 10.1097/RLU.0000000000004099. Epub 2022 Mar 9. PMID: 35261359.	Case report in the field of interest
Yao L, Zhao L, Pang Y, Shang Q, Chen H. Increased 68Ga-FAPI Uptake in Ankylosing Spondylitis in a Patient With Rectal Cancer. Clin Nucl Med. 2022 Feb 1;47(2):176-178. doi: 10.1097/RLU.0000000000003798. PMID: 34269721.	Case report in the field of interest
Liu H, Yang X, Fan D, Lv T, Chen Y. Mesenteric Inflammatory Myofibroblastic Tumor on 68Ga-FAPI PET/CT. Clin Nucl Med. 2021 Dec 1;46(12):1026-1027. doi: 10.1097/RLU.0000000000003797. PMID: 34238804.	Case report in the field of interest
Shou Y, Xue Q, Yuan J, Zhao J. ⁶⁸Ga-FAPI-04 PET/MR is helpful in differential diagnosis of pancreatitis from pancreatic malignancy compared to ¹⁸F-FDG PET/CT: a case report. Eur J Hybrid Imaging. 2021 Jun 15;5(1):12. doi: 10.1186/s41824-021-00106-1. PMID: 34181149; PMCID: PMC8218089	Case report in the field of interest
Linz C, Brands RC, Kertels O, Dierks A, Brumberg J, Gerhard-Hartmann E, Hartmann S, Schirbel A, Serfling S, Zhi Y, Buck AK, Kübler A, Hohm J, Lapa C, Kircher M. Targeting fibroblast activation protein in newly diagnosed squamous cell carcinoma of the oral cavity - initial experience and comparison to [¹⁸F]FDG PET/CT and MRI. Eur J Nucl Med Mol Imaging. 2021 Nov;48(12):3951-3960. doi: 10.1007/s00259-021-05422-z. Epub 2021 May 29.	Original article not in the field of interest

PMID: 34050405; PMCID: PMC8484183.	
Wu S, Pang Y, Zhao L, Zhao L, Chen H. 68Ga-FAPI PET/CT Versus 18F-FDG PET/CT for the Evaluation of Disease Activity in Takayasu Arteritis. Clin Nucl Med. 2021 Oct 1;46(10):847-849. doi: 10.1097/RLU.0000000000003692. PMID: 34028415.	Case report in the field of interest
Luo Y, Pan Q, Yang H, Li F, Zhang F. Inflammatory Arthritis Induced by Anti-Programmed Death-1 Shown in 68Ga-FAPI PET/CT in a Patient With Esophageal Carcinoma. Clin Nucl Med. 2021 May 1;46(5):431-432. doi: 10.1097/RLU.0000000000003608. PMID: 33782307.	Case report not in the field of interest
Iking J, Staniszewska M, Kessler L, Klose JM, Lückerath K, Fendler WP, Herrmann K, Rischpler C. Imaging Inflammation with Positron Emission Tomography. Biomedicines. 2021 Feb 19;9(2):212. doi: 10.3390/biomedicines9020212. PMID: 33669804; PMCID: PMC7922638.	Review not in the field of interest
Sharma P, Singh SS, Gayana S. Fibroblast Activation Protein Inhibitor PET/CT: A Promising Molecular Imaging Tool. Clin Nucl Med. 2021 Mar 1;46(3):e141-e150. Doi: 10.1097/RLU.0000000000003489. PMID: 33351507.	Review not in the field of interest
Hicks RJ, Roselt PJ, Kallur KG, Tothill RW, Mileshkin L. FAPI PET/CT: Will It End the Hegemony of ¹⁸F-FDG in Oncology? J Nucl Med. 2021 Mar;62(3):296-302. Doi: 10.2967/jnumed.120.256271. Epub 2020 Dec 4. PMID: 33277397.	Editorial not in the field of interest
Serfling S, Zhi Y, Schirbel A, Lindner T, Meyer T, Gerhard-Hartmann E, Lapa C, Hagen R, Hackenberg S, Buck AK, Scherzad A. Improved cancer detection in Waldeyer's tonsillar ring by ⁶⁸Ga-FAPI PET/CT imaging. Eur J Nucl Med Mol Imaging. 2021 Apr;48(4):1178-1187. doi: 10.1007/s00259-020-05055-8. Epub 2020 Oct 15. PMID: 33057927; PMCID: PMC8041699.	Original article not in the field of interest
Chen H, Zhao L, Ruan D, Pang Y, Hao B, Dai Y, Wu X, Guo W, Fan C, Wu J, Huang W, Lin Q, Sun L, Wu H. Usefulness of [⁶⁸Ga]Ga-DOTA-FAPI-04 PET/CT in patients presenting with inconclusive [¹⁸F]FDG PET/CT findings. Eur J Nucl Med Mol Imaging. 2021 Jan;48(1):73-86. doi: 10.1007/s00259-020-04940-6. Epub 2020 Jun 25. PMID: 32588089.	Original article not in the field of interest
Luo Y, Pan Q, Zhang W, Li F. Intense FAPI Uptake in Inflammation May Mask the Tumor Activity of Pancreatic Cancer in 68Ga-FAPI PET/CT. Clin Nucl Med. 2020 Apr;45(4):310-311. doi:	Case report in the field of interest

10.1097/RLU.0000000000002914. PMID: 31977474.	
Gu B, Luo Z, He X, Wang J, Song S. 68Ga-FAPI and 18F-FDG PET/CT Images in a Patient With Extrapulmonary Tuberculosis Mimicking Malignant Tumor. Clin Nucl Med. 2020 Nov;45(11):865-867. doi: 10.1097/RLU.0000000000003279. PMID: 32969904; PMCID: PMC7531500.	Case report in the field of interest
Xu T, Zhao Y, Ding H, Cai L, Zhou Z, Song Z, Chen Y. [68Ga]Ga-DOTA-FAPI-04 PET/CT imaging in a case of prostate cancer with shoulder arthritis. Eur J Nucl Med Mol Imaging. 2021 Apr;48(4):1254-1255. doi: 10.1007/s00259-020-05028-x. Epub 2020 Sep 8. PMID: 32901354	Case report in the field of interest
Song Y, Qin C, Liu F, Lan X. Fibrous Dysplasia Mimicking Skeletal Metastasis on 68Ga-FAPI PET Imaging. Clin Nucl Med. 2021 Sep 1;46(9):774-775. doi: 10.1097/RLU.0000000000003671. PMID: 33883497	Case report in the field of interest
Pan Q, Luo Y, Zhang W. Idiopathic Retroperitoneal Fibrosis With Intense Uptake of 68Ga-Fibroblast Activation Protein Inhibitor and 18F-FDG. Clin Nucl Med. 2021 Feb 1;46(2):175-176. doi: 10.1097/RLU.0000000000003402. PMID: 33208623.	Case report in the field of interest
Qin C, Yang L, Ruan W, Shao F, Lan X. Immunoglobulin G4-Related Sclerosing Cholangitis Revealed by 68Ga-FAPI PET/MR. Clin Nucl Med. 2021 May 1;46(5):419-421. doi: 10.1097/RLU.0000000000003552. PMID: 33630803	Case report in the field of interest
Luo Y, Pan Q, Yang H, Peng L, Zhang W, Li F. Fibroblast Activation Protein-Targeted PET/CT with 68Ga-FAPI for Imaging IgG4-Related Disease: Comparison to 18F-FDG PET/CT. J Nucl Med. 2021 Feb;62(2):266-271. doi: 10.2967/jnumed.120.244723. Epub 2020 Jun 8. PMID: 32513902	Case report in the field of interest
Luo Y, Pan Q, Xu H, Zhang R, Li J, Li F. Active uptake of 68Ga-FAPI in Crohn's disease but not in ulcerative colitis. Eur J Nucl Med Mol Imaging. 2021 May;48(5):1682-1683. doi: 10.1007/s00259-020-05129-7. Epub 2020 Nov 27. PMID: 33247327.	Case report in the field of interest
Liu H, Yang X, Wang Y, Wang P, Chen Y. 68Ga-FAPI PET/CT Imaging of Graves Ophthalmopathy in a Patient With Esophageal Cancer. Clin Nucl Med. 2021 Nov 1;46(11):938-939. doi: 10.1097/RLU.0000000000003703. PMID: 34028423	Case report in the field of interest

Liu H, Wang Y, Zhang W, Cai L, Chen Y. Elevated 68Ga-FAPI Activity in Splenic Hemangioma and Pneumonia. Clin Nucl Med. 2021 Aug 1;46(8):694-696. doi: 10.1097/RLU.0000000000003638. PMID: 33826568	Case report in the field of interest
Liu H, Chen Z, Yang X, Fu W, Chen Y. Increased 68Ga-FAPI Uptake in Chronic Cholecystitis and Degenerative Osteophyte. Clin Nucl Med. 2021 Jul 1;46(7):601-602. doi: 10.1097/RLU.0000000000003621. PMID: 33782317	Case report in the field of interest
Lin M, Xue Q, You X, Yao S, Miao W. Cerebral Venous Sinus Thrombosis Caused by Neuro-Behçet Disease Accidentally Detected by 68Ga-FAPI PET/CT. Clin Nucl Med. 2021 Dec 1;46(12):1028-1029. doi: 10.1097/RLU.0000000000003790. PMID: 34238803.	Case report in the field of interest
Liu H, Yang X, Liu L, Lei L, Wang L, Chen Y. Clinical Significance of Diffusely Increased Uptake of 68Ga-FAPI in Thyroid Gland. Front Med (Lausanne). 2021 Nov 23;8:782231. doi: 10.3389/fmed.2021.782231. PMID: 34888332; PMCID: PMC8649924.	Case report in the field of interest
Hotta M, Sonni I, Benz MR, Gafita A, Bahri S, Shuch BM, Yu R, Liu ST, Czernin J, Calais J. 68Ga-FAPI-46 and 18F-FDG PET/CT in a patient with immune-related thyroiditis induced by immune checkpoint inhibitors. Eur J Nucl Med Mol Imaging. 2021 Oct;48(11):3736-3737. doi: 10.1007/s00259-021-05373-5. Epub 2021 Apr 29. PMID: 33914106	Case report in the field of interest
Hao B, Wu X, Pang Y, Sun L, Wu H, Huang W, Chen H. [18F]FDG and [68Ga]Ga-DOTA-FAPI-04 PET/CT in the evaluation of tuberculous lesions. Eur J Nucl Med Mol Imaging. 2021 Feb;48(2):651-652. doi: 10.1007/s00259-020-04941-5. Epub 2020 Jul 8. PMID: 32643006.	Case report in the field of interest
Can C, Gündoğan C, Güzel Y, Kaplan İ, Kömek H. 68Ga-FAPI Uptake of Thyroiditis in a Patient With Breast Cancer. Clin Nucl Med. 2021 Aug 1;46(8):683-685. doi: 10.1097/RLU.0000000000003637. PMID: 33826569.	Case report in the field of interest
Jiang C, Song S. 68Ga-FAPI and 18F-FDG PET/CT in Perineum Extramammary Paget Disease. Clin Nucl Med. 2021 Apr 1;46(4):342-344. doi:	Case report in the field of interest

10.1097/RLU.0000000000003523. PMID: 33577199.	
Zheng J, Chen H, Lin K, Yao S, Miao W. [68Ga]Ga-FAPI and [18F]FDG PET/CT images in a patient with juvenile polymyositis. Eur J Nucl Med Mol Imaging. 2021 Jun;48(6):2051-2052. doi: 10.1007/s00259-020-05185-z. Epub 2021 Jan 18. PMID: 33462628.	Case report in the field of interest
Zhou Y, He J, Chen Y. 68Ga-FAPI PET/CT imaging in a patient with thyroiditis. Endocrine. 2021 Aug;73(2):485-486. doi: 10.1007/s12020-021-02605-4. Epub 2021 Jan 15. PMID: 33449295.	Case report in the field of interest
Pan Q, Luo Y, Zhang W. Recurrent Immunoglobulin G4-Related Disease Shown on 18F-FDG and 68Ga-FAPI PET/CT. Clin Nucl Med. 2020 Apr;45(4):312-313. doi: 10.1097/RLU.0000000000002919. PMID: 31977476.	Case report in the field of interest
Zhao L, Gu J, Fu K, Lin Q, Chen H. 68Ga-FAPI PET/CT in Assessment of Liver Nodules in a Cirrhotic Patient. Clin Nucl Med. 2020 Oct;45(10):e430-e432. doi: 10.1097/RLU.0000000000003015. PMID: 32332301.	Case report in the field of interest
Luo Y, Pan Q, Zhang W. IgG4-related disease revealed by 68Ga-FAPI and 18F-FDG PET/CT. Eur J Nucl Med Mol Imaging. 2019 Nov;46(12):2625-2626. doi: 10.1007/s00259-019-04478-2. Epub 2019 Aug 13. PMID: 31410541.	Case report in the field of interest
Vorster M. Gallium-68 Labelled Radiopharmaceuticals for Imaging Inflammatory Disorders. Semin Nucl Med. 2023 Mar;53(2):199-212. doi: 10.1053/j.semnuclmed.2022.08.005.	Review in the field of interest
Zhang M, Quan W, Zhu T, Feng S, Huang X, Meng H, Du R, Zhu Z, Qu X, Li P, Cui Y, Shi K, Yan X, Zhang R, Li B. [68Ga]Ga-DOTA-FAPI-04 PET/MR in patients with acute myocardial infarction: potential role of predicting left ventricular remodeling. Eur J Nucl Med Mol Imaging. 2023 Feb;50(3):839-848. doi: 10.1007/s00259-022-06015-0.	Original article not in the field of interest
Qiao P, Wang Y, Zhu K, Zheng D, Song Y, Jiang D, Qin C, Lan X. Noninvasive Monitoring of Reparative Fibrosis after Myocardial Infarction in Rats Using 68Ga-FAPI-04 PET/CT. Mol Pharm. 2022 Nov 7;19(11):4171-4178. doi: 10.1021/acs.molpharmaceut.2c00551.	Preclinical study
Lyu Z, Han W, Zhao H, Jiao Y, Xu P, Wang Y, Shen Q,	Original article not in the field of interest

Yang S, Zhao C, Tian L, Fu P. A clinical study on relationship between visualization of cardiac fibroblast activation protein activity by Al18F-NOTA-FAPI-04 positron emission tomography and cardiovascular disease. Front Cardiovasc Med. 2022 Aug 22;9:921724. doi: 10.3389/fcvm.2022.921724.	
Da Pieve C, Costa Braga M, Turton DR, Valla FA, Cakmak P, Plate KH, Kramer-Marek G. New Fully Automated Preparation of High Apparent Molar Activity 68Ga-FAPI-46 on a Trasis AiO Platform. Molecules. 2022 Jan 20;27(3):675. doi: 10.3390/molecules27030675.	Original article not in the field of interest
Diekmann J, Koenig T, Thackeray JT, Derlin T, Czerner C, Neuser J, Ross TL, Schäfer A, Tillmanns J, Bauersachs J, Bengel FM. Cardiac Fibroblast Activation in Patients Early After Acute Myocardial Infarction: Integration with MR Tissue Characterization and Subsequent Functional Outcome. J Nucl Med. 2022 Sep;63(9):1415-1423. doi: 10.2967/jnumed.121.263555	Original article not in the field of interest
Kessler L, Kupusovic J, Ferdinandus J, Hirmas N, Umutlu L, Zarrad F, Nader M, Fendler WP, Totzeck M, Wakili R, Schlosser T, Rassaf T, Rischpler C, Siebermair J. Visualization of Fibroblast Activation After Myocardial Infarction Using 68Ga-FAPI PET. Clin Nucl Med. 2021 Oct 1;46(10):807-813. doi: 10.1097/RLU.0000000000003745.	Original article not in the field of interest
Xie B, Wang J, Xi XY, Guo X, Chen BX, Li L, Hua C, Zhao S, Su P, Chen M, Yang MF. Fibroblast activation protein imaging in reperfused ST-elevation myocardial infarction: comparison with cardiac magnetic resonance imaging. Eur J Nucl Med Mol Imaging. 2022 Jul;49(8):2786-2797. doi: 10.1007/s00259-021-05674-9.	Original article not in the field of interest
Zhu W, Guo F, Wang Y, Ding H, Huo L. 68Ga-FAPI-04 Accumulation in Myocardial Infarction in a Patient With Neuroendocrine Carcinoma. Clin Nucl Med. 2020 Dec;45(12):1020-1022. doi: 10.1097/RLU.0000000000003334.	Case report not in the field of interest
Notohamiprodjo S, Nekolla SG, Robu S, Villagran Asiares A, Kupatt C, Ibrahim T, Laugwitz KL, Makowski MR, Schwaiger M, Weber WA, Varasteh Z. Imaging of cardiac fibroblast activation in a patient after acute myocardial infarction using 68Ga-FAPI-04. J Nucl Cardiol. 2022 Oct;29(5):2254-2261. doi: 10.1007/s12350-021-02603-z.	Original article not in the field of interest
Siebermair J, Köhler MI, Kupusovic J, Nekolla SG, Kessler L, Ferdinandus J, Guberina N, Stuschke M, Grafe H, Siveke JT, Kochhäuser S, Fendler WP,	Original article not in the field of interest

Totzeck M, Wakili R, Umutlu L, Schlosser T, Rassaf T, Rischpler C. Cardiac fibroblast activation detected by Ga-68 FAPI PET imaging as a potential novel biomarker of cardiac injury/remodeling. J Nucl Cardiol. 2021 Jun;28(3):812-821. doi: 10.1007/s12350-020-02307-w	
Heckmann MB, Reinhardt F, Finke D, Katus HA, Haberkorn U, Leuschner F, Lehmann LH. Relationship Between Cardiac Fibroblast Activation Protein Activity by Positron Emission Tomography and Cardiovascular Disease. Circ Cardiovasc Imaging. 2020 Sep;13(9):e010628. doi: 10.1161/CIRCIMAGING.120.010628.	Original article not in the field of interest
Varasteh Z, Mohanta S, Robu S, Braeuer M, Li Y, Omidvari N, Topping G, Sun T, Nekolla SG, Richter A, Weber C, Habenicht A, Haberkorn UA, Weber WA. Molecular Imaging of Fibroblast Activity After Myocardial Infarction Using a 68Ga-Labeled Fibroblast Activation Protein Inhibitor, FAPI-04. J Nucl Med. 2019 Dec;60(12):1743-1749. doi: 10.2967/jnumed.119.226993	Original article not in the field of interest
